# Risperidone and NAP protect cognition and normalize gene expression in a schizophrenia mouse model

**DOI:** 10.1038/srep16300

**Published:** 2015-11-10

**Authors:** Sinaya Vaisburd, Zeev Shemer, Adva Yeheskel, Eliezer Giladi, Illana Gozes

**Affiliations:** 1 The Lily and Avraham Gildor Chair for the Investigation of Growth Factors, The Elton Laboratory for Neuroendocrinology, Department of Human Molecular Genetics and Biochemistry, Sackler Faculty of Medicine, Sagol School of Neuroscience and Adams Super Center for Brain Studies, Tel Aviv University 69978, Israel.; 2 The Bioinformatics Unit, George S. Wise Faculty of Life Sciences, Tel Aviv University 69978, Israel

## Abstract

Mutated disrupted in schizophrenia 1 (DISC1), a microtubule regulating protein, leads to schizophrenia and other psychiatric illnesses. It is hypothesized that microtubule stabilization may provide neuroprotection in schizophrenia. The **NAP** (**NAP**VSIPQ) sequence of activity-dependent neuroprotective protein (ADNP) contains the SxIP motif, microtubule end binding (EB) protein target, which is critical for microtubule dynamics leading to synaptic plasticity and neuroprotection. Bioinformatics prediction for FDA approved drugs mimicking SxIP-like motif which displace NAP-EB binding identified Risperidone. Risperidone or NAP effectively ameliorated object recognition deficits in the mutated DISC1 mouse model. NAP but not Risperidone, reduced anxiety in the mutated mice. Doxycycline, which blocked the expression of the mutated DISC1, did not reverse the phenotype. Transcripts of Forkhead-BOX P2 (Foxp2), a gene regulating DISC1 and associated with human ability to acquire a spoken language, were increased in the hippocampus of the DISC1 mutated mice and were significantly lowered after treatment with NAP, Risperidone, or the combination of both. Thus, the combination of NAP and standard of care Risperidone in humans may protect against language disturbances associated with negative and cognitive impairments in schizophrenia.

Schizophrenia is a psychiatric disorder that affects about 1% of the world’s population. The social effects of schizophrenia are well known, but the nature of the disease still remains vague. Schizophrenia is known to be associated with neuronal dysfunction involving the cytoskeletal system.

DISC1 (disrupted in schizophrenia 1) located on chromosome 1, was identified in a large Scottish familial pedigree with major psychiatric disorders and schizophrenia^1^. The particular impairment that has been found was a balanced chromosomal translocation breakpoint of [(1: 11) (q42.1; q14.3)]; the breakpoint was in the middle of an open reading frame region for the gene, which probably yielded an expression of an abnormal-truncated protein. Normal DISC1 is expressed in the hippocampal dentate gyrus, cerebral cortex, hypothalamus, amygdala, cerebellum, and olfactory bulbs^2^. The truncated, human DISC1 (hDISC1) loses its normal localization and association with its interacting proteins such as the microtubules – MTs and MT-associated proteins. This results in decreased complexity of dendritic arbors and decreased neurite outgrowth^3^, as seen in animal-derived cellular models^4^. Dendritic abnormalities were also found in post-mortem brain samples of patients with schizophrenia^5^. Other psychiatric illnesses such as clinical depression and bipolar disorder have also been associated to the DISC1 mutation/ truncation^6^. Mice expressing the mutant human DISC1 may provide a reliable model for psychiatric illnesses such as schizophrenia with schizophrenia-like symptoms including deficits in memory, although it should be borne in mind that the proportion of individuals with DISC1 mutations suffering from schizophrenia is rather small^7^.

NAP (davunetide, NAPVSIPQ) is a fragment of activity-dependent neuroprotective protein (ADNP). NAP has been proven to be a neuroprotective agent by interacting with its target, the MT end- binding (EB) proteins through its SIP motif^8^. NAP affects neuronal MTs by their stabilization and by enhancement of the MT-dependent axonal transport^9^,^10^,^11^. The EB protein family that consists of three members (EB1–3) is the core component of the MT plus-end–tracking proteins (+TIPs) machinery which coordinates a network of dynamic proteins on the growing MT plus-ends. The majority of EB-recruited +TIPs, bind to the EBs through a short hydrophobic (S/T) X (I/L) P sequence motif (SxIP). The SxIP motif is a conserved sequence with a certain degree of freedom on the second position [X]^12^. In neurons, EB1 has been involved in axonal transport, whereas EB3 has been characterized as a molecular link between MTs and the actin cytoskeleton. Our hypothesis is that NAP alone and synergistically with other EB3-interacting drugs can effectively improve functional activity in schizophrenia, as tested in the transgenic (Tg) DISC1 mutated mice.

Interestingly, DISC1 has been associated with verbal fluency in schizophrenia^13^ and a recent study revealed a link between DISC1 and Forkhead -BOX P2 (FOXP2), the gene associated with human ability to acquire spoken language, with FOXP2 regulating DISC1 transcripts^14^. The FOXP2 protein modulates transcription, consequently influencing the relative abundances of cellular proteins. Mutations in FOXP2 cause developmental disorders that significantly disrupt speech and language skills^15^,^16^. Although only humans acquire spoken language ability, FOXP2 is well-preserved in animals. Only three amino acids distinguish the human FOXP2 protein from the mouse one. Two of these changes that occurred on the human lineage after separation from the human-chimp common ancestor, are probably responsible for the gap in speaking ability. Recent studies have found that FOXP2 polymorphisms are associated with schizophrenia in human cohorts^17^,^18^,^19^. Moreover, SNPs of the FOXP2 gene were identified to be associated with schizophrenia and major depression within the Chinese population^20^. Thus, the FOXP2 gene might be involved in the language disturbances found in patients with schizophrenia. During the development of the organism, or in response to the internal/external stimuli, FOXP2 modifies the expression levels of different genes in a tissue-specific manner^21^.

Here, we set up to evaluate NAP, as a treatment against cognitive deficits and impairments in Foxp2 expression in a DISC1 mutated mouse model for schizophrenia. Controls included doxycycline treatment which blocked the expression of the mutated gene and Risperidone, a frequently used neuroleptic.

## Results

### Risperidone (RIS) is an EB1/EB3 interacting molecule

**Figure 1:**
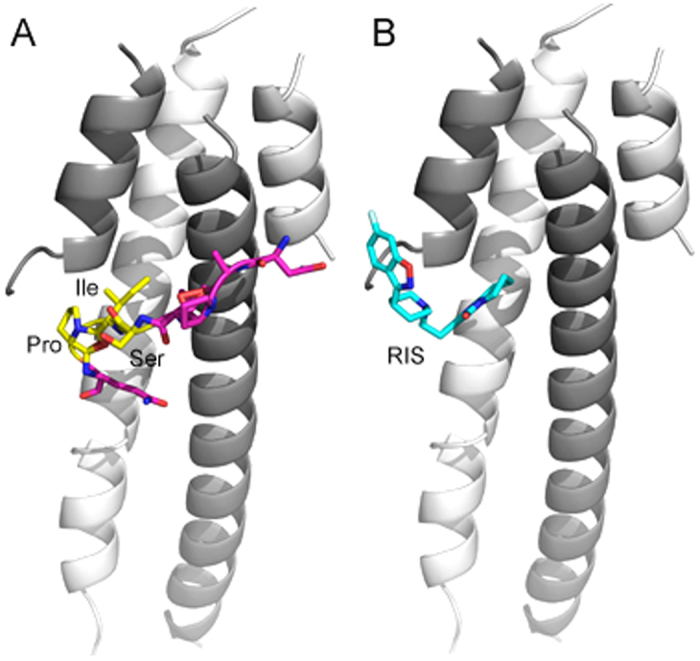
NAP and RIS binding prediction to EB3. (**A**) NAP (NAPVSIPQ) peptide binding to EB3. EB3 dimer is shown as white and gray cartoons (PDB 3TQ7, residues in chain A 197–248 and chain B 204–258). The peptide is shown as purple sticks, while the SIP motif is colored in yellow. The binding orientation was predicted using structural alignment of EB3 (PDB 3TQ7) to EB1-EB3 complex with peptide MACF (PDB 3GJO). (**B**) RIS binding to EB3, as predicted by Swissdock. RIS is shown in cyan sticks. The figure was generated using Pymol.

NAP contains a functional segment SxIP (SIP domain), which has been implicated as a MT end binding protein (EB), interacting domain. Previous experiments in our laboratory showed that NAP binds to EB1 and EB3^22^. Bioinformatics techniques were applied, developing a model based on EB1–3 and NAP protein structure (Fig. 1A). This was followed by screening application on FDA approved drugs directories on Edesign web server^23^,^24^. RIS was identified as an EB1/EB3 interacting molecule (Fig. 1B).

### Risperidone (RIS) competes with NAP binding on EB3

**Figure 2:**
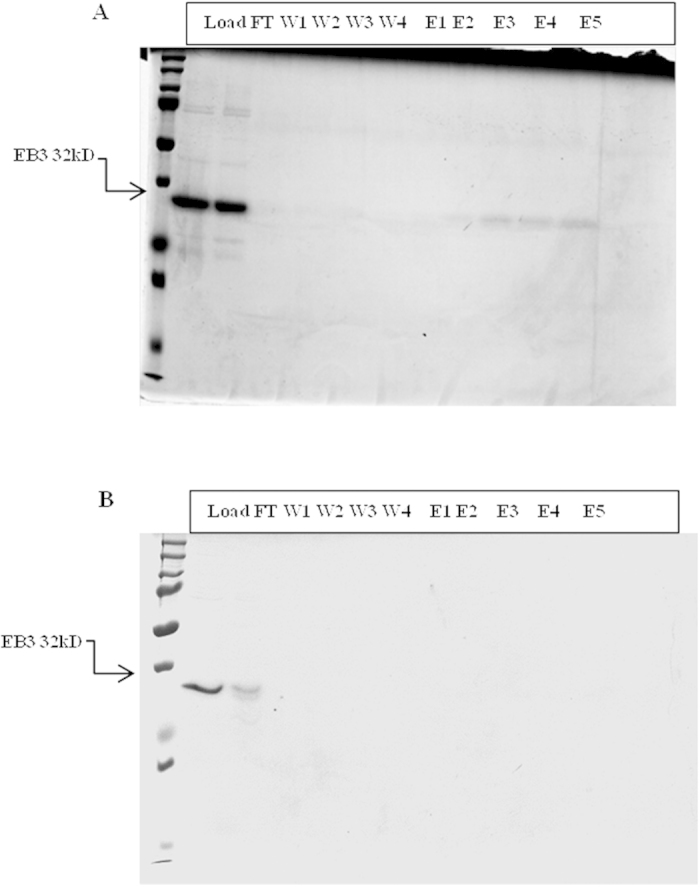
RIS prevents EB3–NAP (CKKKGGNAPVSIPQ) interaction. Competitive affinity chromatography column with NAP bound to SulfoLink® coupling resin loaded with recombinant EB3 with or without RIS. Samples were separated by electrophoresis on SDS polyacrylamide gel (12%) followed by staining with Coomassie Brilliant Blue (CBB). The stained gel results are shown. (**A**) Binding to NAP (control column): recombinant EB3 (3 mg) was loaded, EB3 presence in the following fractions: load, flow- through and elution (number 2–5), validates EB3-NAP interaction, with EB3 eluting with high acid concentrations (lanes 3–5). (**B**) RIS (3 mg) and EB3 (3 mg) were loaded together, EB3 presence in the following fractions: flow- through and wash (number 1) and not in the elution fractions indicates that RIS prevented EB3-NAP interaction.

RIS was tested for affinity binding with recombinant EB3 protein. Binding was assessed using competitive affinity chromatography column with CKKKGGNAPVSIPQ (NAP) conjugated to Sulfolink® coupling resin and loaded with recombinant EB3. The SulfoLink® Immobilization Kit for Peptides (Thermo Scientific, Rockford, IL) conjugated to CKKKGGNAPVSIPQ(c-NAP) beads was used^25^. RIS prevented the NAP-EB3 interaction (Fig. 2A w/o RIS; Fig. 2B + RIS). These results suggest that the NAP peptide and RIS share a similar binding site, confirming the bioinformatics results.

### The expression of the DISC1 mutated protein inhibited by doxycycline (DOX) treatment

**Figure 3:**
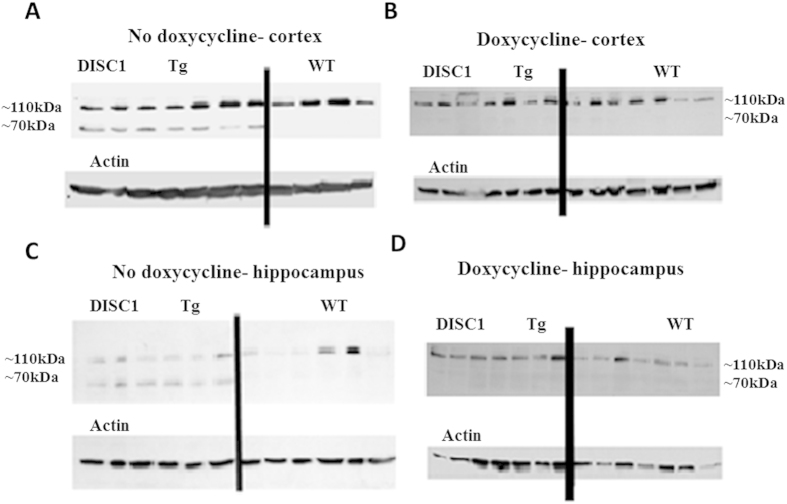
Doxycycline inhibits transgene synthesis. Western blot results for DISC1 protein in DD-treated Tg mice vs. DD-treated WT mice in the prefrontal cortex and the hippocampus area. The band that represents the normal DISC1 protein has a molecular weight of ~110 kDa and the band underneath it represents the truncated human mutant DISC1 with a molecular weight of ~70k Da. The expression of DISC1 protein in mice without DOX treatment in the cortex area (**A**) and the hippocampus area (**C**) is presented. Only the truncated mutant DISC1 band is presented in the Tg mice group, this verifies the difference between the Tg mice, which express the human mutant protein and the WT mice which express only the normal DISC1 protein. The expression of DISC1 protein in mice with DOX treatment in the cerebral cortex area (**B**) and the hippocampus area (**D**) is presented. In figures B and D, only the normal DISC1 band is presented, which confirms that DOX treatment blocked the expression of the human mutant DISC1 protein.

To obtain an overview of DISC1 expression and the inhibitory effect of DOX, a semi-quantitative Western blotting approach was used (Fig. 3A–D). Figure 3A,C shows DISC1-like-immunoreactive protein blots in extracts derived from either the prefrontal cortex or the hippocampus of mice without DOX treatment. A clear difference was observed between the vehicle-treated Tg mice and the vehicle-treated wild-type (WT) littermates. The difference is in the form of a second lower band that appeared in the vehicle-treated Tg mouse group. This lower protein band represents the truncated human mutant DISC1 protein that was produced by the vehicle-treated Tg mouse group. It means that the first part of the inducible DISC1 transgenic mouse model was working, i.e., the Tg mice expressed the human mutant DISC1 protein which has a negative dominant effect on the normal DISC1 protein^6^,^3^. Figure 3B,D shows the expression of the DISC1 protein in the hippocampus and prefrontal cortex area, in mice exposed to DOX treatment, showing no visible difference in the expression of the truncated second DISC1 band, between Tg and WT mice. These results imply that the second part of the inducible DISC1 transgenic mice model was working as well. Thus, DOX treatment led to a cessation in the expression of the human mutant DISC1 protein.

### NAP or Risperidone (RIS) increase cognitive function in the DISC1 mutated mice

**Figure 4:**
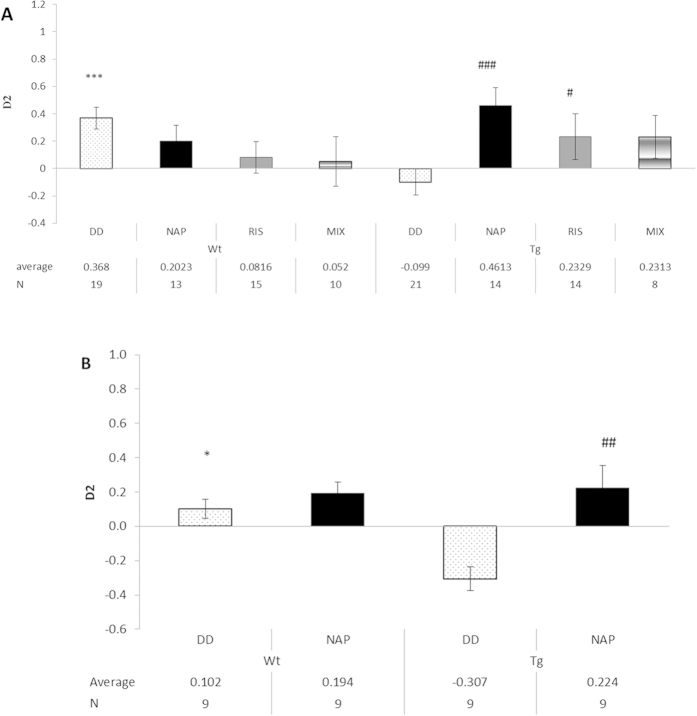
Object recognition, short retention memory test: effects of NAP and DOX. (**A**) Animal performance in the object recognition memory test is shown (N/group indicated on the figure). Data are expressed as mean (±SEM) total time spent exploring all objects designated by relative discrimination index (D2) in Phase II that includes one object that was presented during Phase I, and one novel object. Two-way ANOVA showed no significant effect of treatment (F (3,105) = 1.103, P = 0.351) and no significant effect of genotype (F(1,105) = 0.099, P = 0.753), there was a statistically significant interaction effect between treatment and genotype (P = 0.006). Fisher’s LSD post hoc test revealed that the Tg mice under DD (vehicle) treatment showed significant deficits in recognizing the novel object and seemed to prefer the familiar object in comparison to control WT mice under DD (vehicle) treatment (***p < 0.002). NAP and RIS treatment significantly improved object recognition/discrimination in the Tg mice to a control WT level (^###^p < 0.001, ^#^P = 0.043 respectively). (**B**) DOX treatment was ineffective, i.e. the DOX-Tg mice preferred the known/familiar object in contrast to the DOX-WT mice and the DOX-Tg group was similar to the non-DOX- treated Tg group, shown in A. (DOX-Tg vs. DOX-WT – *p < 0.05, NAP-treated DOX-Tg vs. DoX-Tg, ^##^p < 0.01).

Novel object recognition in rodents is analogous in some ways to human declarative (episodic) memory, one of the cognitive domains which is abnormal in schizophrenia^26^. Our previous experiments in the MT associated protein 6 –deficient mouse model in schizophrenia showed no significant learning deficits in the Morris water maze, and only a significant memory deficit in the probe trial^27^. In contrast, significant and reproducible results were obtained with the object recognition test^27^,^28^, and hence this test was chosen for use here. However, a subset of mice was also tested in the Morris water maze, indicating essentially no genotype effect on learning and short term spatial memory and no deficits in the probe trial (Suppl. Fig. 1A,B). Furthermore, no differences were noted in the open field test (Suppl. Fig. 2). In the novel object recognition, in the memory retention test (Fig. 4), DISC1 mice (Tg) explored the novel object for a significantly shorter time period than the control WT under DD (vehicle) treatment (discrimination index, −0.099 vs. 0.368; ***p < 0.002, for genotype effect) demonstrating a degraded memory capability. The Tg NAP-treated mice spent significantly longer time periods with the novel object compared to the Tg DD (vehicle)-treated mice (discrimination index, 0.4613 vs.−0.099; ^###^p < 0.001). The RIS-treated mice showed a significant increase in the interaction time with the novel object in comparison to Tg mice under DD (vehicle) treatment (discrimination index, 0.2329 vs.−0.099; ^#^p = 0.043). MIX – treated mice did not demonstrate an enhancement in discrimination toward the novel object compared to Tg mice under DD (vehicle) treatment (discrimination index, 0.2313 vs.−0.099; p = 0.088), possibly due to the group variability, requiring a larger group of test mice. However, the results of the MIX and the RIS groups were almost identical, suggesting no added value for the NAP treatment, which was apparently more efficient on its own (Fig. 4A). It should be added that RIS has shown protective activity before in the novel object recognition test in rats, for example, after post-weaning social isolation^29^ and following neonatal intra-hippocampal injection of lipopolysaccharides^30^. Finally, in our current studies, the drug-treatment results contrasted DOX-treatment results which were ineffective and the DOX-treated Tg group was similar to the non-treated Tg group (Fig. 4B). Additional behavioral tests suggested also defective social memory and future experiments with large experimental groups could utilize these tests (Suppl. Fig. 3).

### NAP, but not Risperidone (RIS) normalizes behavior in the elevated plus maze

**Figure 5:**
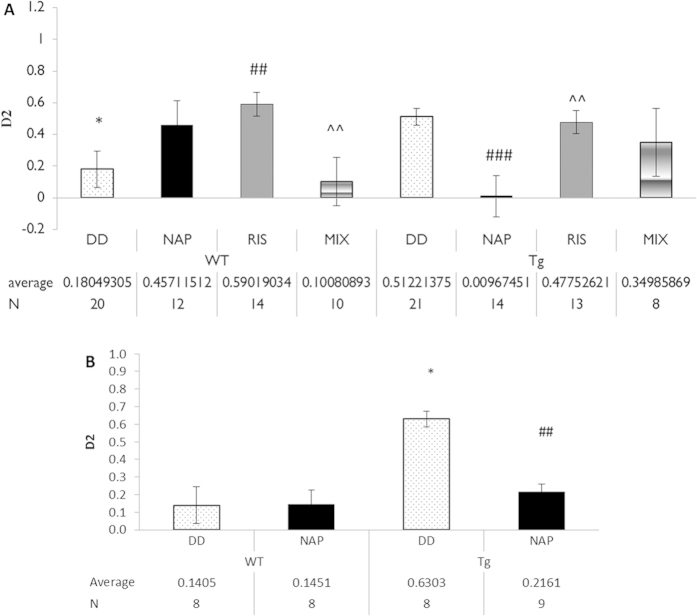
Elevated plus-maze test: effects of NAP and DOX. (**A**) The number of total arm entries (counts) and time spent in the open-arms for 5 min are presented (N/group indicated on the figure).Data are expressed as mean (±SEM) total time spent exploring all objects designated by relative discrimination index (D2) = (time in close arms – time in open arms)/(time in close arms + time in open arms). Two way ANOVA showed no significant effect of genotype (F (1,105) = 0.295, P = 0.588) and a statistically significant effect of treatment (F (3,112) = 0.012, P = 0.012) as well as an interaction between genotype and treatment (P = 0.021). Post hoc Fisher’s LSD test revealed a significant difference between the D2 index between Tg mice under DD (vehicle) treatment and control WT mice under DD (vehicle) treatment (*p = 0.016), NAP significantly reduced anxiety within Tg mice compared to DD treatment (^###^p = 0.001) and, RIS (^^p = 0.006). WT mice under RIS treatment behaved unlike control WT mice, exhibiting a significantly higher index ratio in closed/open arm than the WT DD (vehicle)-treated mice and mix treated mice (^##^p = 0.009, ^^p = 0.008, respectively). (**B**) DOX treatment was ineffective and the DOX-treated group was similar to the non-treated group i.e. the DOX-Tg mice spent more time in the closed arms of the maze compared to the DOX-WT mice and the DOX-Tg group was similar to the non-DOX- treated Tg group, shown in A. (DOX-Tg vs. DOX-WT – *p < 0.05, NAP-treated DOX-Tg vs. DoX-Tg, ^##^p < 0.01).

In order to evaluate the anxiety related behavior, an elevated plus maze test was conducted (Fig. 5). The ratio between the time spent in the closed arms to the time spent in the open arms, was considerably higher within the Tg mice treated with DD in comparison to the WT mice treated with DD (vehicle), (discrimination index – D2, 0.512 vs. 0.180; *p = 0.016, for genotype effect). The Tg mice showed reduced exploratory behavior (less time spent in the open space) indicating an increase in anxious behavior. RIS- treated WT mice exhibited a significantly higher index ratio in closed/open arm than the WT DD (vehicle)-treated mice (discrimination index, 0.590 vs. 0.180; ^##^p = 0.009). WT mice treated with MIX behaved like the control WT mice, significantly differing from the RIS treatment group (discrimination index, 0.1008 vs. 0.590 ^^p = 0.008). There was statistically significant difference within the DISC1 Tg mouse group. The NAP-treated Tg mice showed an even lower discrimination index compared to DD (vehicle) and RIS treated mice (discrimination index, 0.009 vs. 0.512; ^###^p = 0.001and 0.009 vs. 0.477; ^^p = 0.006, respectively). This suggests that NAP treatment reduced the anxiety state of the Tg mice. MIX treatment (combination of NAP and RIS) was insufficient to reduce anxiety state compared to NAP treatment alone. Together, the results suggest that 1] RIS treatment in WT mice increased anxiety, which was protected by NAP treatment and that 2] the DISC1 genotype showed increased anxiety as compared to WT, which was normalized by NAP treatment but not by RIS treatment. Furthermore, 3] the anxiety state of the mutated DISC1 genotype was not improved by the MIX treatment (i.e. the mix treatment was ineffective), (Fig. 5A). DOX treatment was ineffective regarding the transgene influence on anxiety/exploratory behavior. Thus, even after DOX injection, the mutated DISC1 mice remained anxious (i.e. spent more time in the closed arms of the maze) and NAP treatment ameliorated this behavior (Fig. 5B).

#### Foxp2 expression is significantly increased in the hippocampus of DISC1-mutated mice: normalization by NAP or Risperidone (RIS) treatment

**Figure 6:**
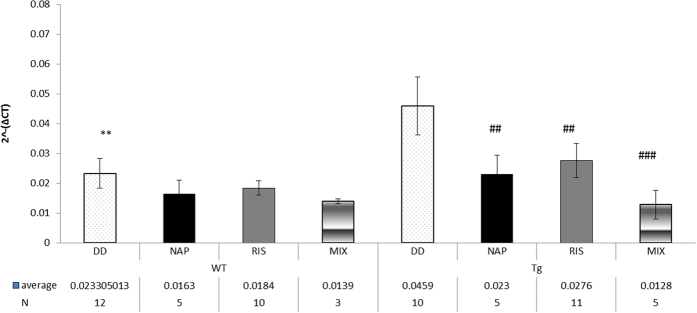
The expression level of Foxp2 gene in DISC1 mice. Expression in the hippocampus area compared among the different groups (N/group indicated on the figure). The two-way ANOVA test showed no significant effect of genotype (F(1,53) = 3.189, P = 0.08), a statistically significant treatment effect (F(3,53) = 3.371, P = 0.015). No significant interaction between genotype and treatment (P = 0.382). Post hoc Fisher’s LSD test revealed significantly higher expression levels of Foxp2, in the DD-treated Tg mice in comparison to the DD-treated WT mice, i.e. a significant model (**P = 0.006), all treatment groups in the Tg mice showed significantly lower expression level of Foxp2 compared to Tg DD-treated mice (NAP, RIS ^##^p = 0.026 and MIX ^###^p = 0.002).

Given the general ineffective effect of DOX treatment in mice after birth, it was assumed that critical effects of the DISC1 mutations are associated with developmental deficits. We thus chose to evaluate the expression of FOXP2, a gene critical for brain development, language acquisition and regulation of DISC1 expression^14^. Quantitative real-time PCR was used to compare the hippocampal gene expression levels of Foxp2 within the different mouse groups. RT-PCR assay of Foxp2 gene expression in the hippocampus showed a significant increase of Foxp2 transcripts in Tg mice under DD treatment in comparison to the WT mice under DD treatment (Fig. 6, 0.04 vs. 0.023; **p = 0.006). All the Tg mouse treatment groups showed significantly lower expression level of Foxp2 compared to DD -treated Tg mice. Similar studies were performed in the cerebral cortex showing no difference in the various treatment groups (data not shown). In detail, the NAP-treated Tg mice exhibited a significantly lower level of Foxp2 RNA than the DD-treated Tg mice (0.023 vs. 0.04; ^#^p = 0.026). RIS-treated mice showed less Foxp2 RNA expression than DD treatment (0.027 vs. 0.04; ^#^p = 0.026). The MIX treated mice displayed significantly lower Foxp2 RNA expression than the DD treated mice (0.01 vs. 0.04; ^##^p = 0.002), which showed that the combination of RIS + NAP normalized Foxp2 expression (Fig. 6).

## Discussion

The results of the current study demonstrate that NAP and RIS effectively ameliorated some behavioral deficits in the Tg mutant DISC1 mouse model. NAP-associated behavioral protection was different compared to RIS, and the combination did not result in an additive effect. Both NAP and RIS protected the object recognition task, however, NAP, unlike RIS, protected against anxiety (reduced exploratory behavior). Controls included DOX treatment, at the adult state, which blocked the expression of the mutated DISC1 gene, nevertheless DOX application did not reverse the phenotype, suggesting that DISC1 expression is important for brain development and the mutated DISC1 expression results in permanent damage. Additionally, we found an increase in Foxp2 transcripts in the hippocampus of the DISC1 schizophrenia mouse model, which was significantly lowered after treatment with NAP, RIS, or the combination of NAP with RIS, raising the possibility that Foxp2 is associated with cognitive functions (object recognition) in these mice.

Antipsychotic medications are considered the standard of care in the treatment of schizophrenia. These medications reduce the positive symptoms of psychosis and prevent their recurrence^31^. However, these drugs are ineffective in the treatment of the negative or cognitive symptoms of schizophrenia. Worth mentioning is that a considerable number of patients with schizophrenia suffer from cognitive decline and poor quality of life^32^. In a phase II clinical trial (~20 subjects/group), patients with schizophrenia who have been treated with NAP (davunetide) showed improvement in daily activities [measured by the UCSD Performance-based Skills Assessment (UPSA)], which indicates protection of functional activity by NAP treatment^33^. The authors concluded that upon effect-size considerations, sample sizes of at least 45–50 subjects/group would be required to obtain significant effects on both MATRICS Consensus Cognitive Battery (MCCB)-rated cognition and UPSA, thus, providing guidance for continued clinical development in schizophrenia. It should be considered that UPSA is a measure of several activities of daily living in human beings, such as the ability to write a check, the ability to ride the public transportation and the ability to comply with medication management. These rely on higher brain functions that are not found in mice and inability to measure those behaviors accurately in mice, may hamper translation of drug candidate into the clinical scenario. Regardless, in our animal studies, we do normalize behaviors in the animal model with NAP treatment, suggestive of potential therapeutic benefit in men, while being completely aware of animal study limitations.

NAP’s active domain that interacts with MT-end binding proteins (EB1 and EB3) is the SxIP motif. Planning our study we looked for drug candidates containing the SxIP motif structure, like small chemical agents that can potentially interact with MTs, as the NAP peptide^22^. RIS was one of those drugs. RIS is a second-generation antipsychotic drug that is used for the treatment of several psychiatric disorders and among other actions, improves the neurocognitive functions in patients with schizophrenia. For example, BL-2010, a drug designed to address cognitive problems in schizophrenia, behaved like RIS in the clinical trial setting^34^. These cognitive effects of RIS in the clinical setting (cited above) agree with our animal findings. The mechanism of action is not entirely understood, however current theories focus mainly on its ability to block dopamine D2 and serotonin 5-HT2A receptors leading to reduction of dopaminergic neurotransmission in the mesolimbic pathway improving the cognitive function in schizophrenia^35^,^36^. We found that treatments with either NAP or RIS significantly improved the Tg DISC1 mouse performance in the memory retention test, uncovering an additional target for RIS activity.

NAP protection against anxiety was observed before, in the elevated plus maze test^37^. Here, we found that NAP treatment reduced the anxiety-like behavior in the Tg mice to the level of the control WT group, this effect was not emulated by RIS treatment, which increased anxiety in the WT group (also protected by NAP treatment). It should be added here that this was somewhat unexpected as RIS may also serve as a compound reducing anxiety, although another rodent study did not show a RIS effect in the elevated plus maze^38^, and the RIS effect may be genotype-dependent, as in fact, seen in our experiment (Fig. 5A). This genotype-dependent assumption was validated in a human trial, i.e. there was a differential response to RIS depending on the genetic background of the healthy human volunteers^39^. The most frequent reaction, in the healthy volunteers was sleepiness, which could perhaps be translated to our mouse finding in the elevated plus maze, preference of the dark arm rather than exploration of the open arm, which is also indicative of alertness.

Further analysis using DOX administration that suppresses hDISC1expression did not reverse the phenotype, which confirms the assumption that permanent brain abnormalities are created throughout development while the MT-based therapy used here, may have increased synaptic plasticity resulting in cognitive plasticity as suggested by our mechanistic published cell culture results^8^. Turning off the expression of the inducible DISC1 mutant by DOX administration at the adult stage, did not revert the phenotype leading to the conclusion that DISC1 is vital for the development.

Our additional goal was to examine if there are expression changes of genes related to development in the Tg DISC1 mice and whether NAP or RIS treatment could regulate those changes. Recent studies showed that FOXP2 gene is important gene in development and brain formation^40^,^41^. Also, an up-regulation of the rodent Foxp2 protein product in both cerebellum (P0, P35) and hippocampus (P35, P56) was identified following a prenatal viral infection, which gives a new insight on up-regulation of this gene at various embryogenic stages^42^. Importantly, a recent study revealed a link between DISC1 and FOXP2 in which the transcription factor, FOXP2, regulates DISC1 transcripts^14^. Real-time PCR RNA expression analysis revealed a significant increase in Foxp2 transcripts in the hippocampus of the DISC1 schizophrenia mouse model, compared to the control group WT mice. The mice that were treated with NAP demonstrated lower levels of Foxp2 transcripts. To the best of our knowledge, this research presents new evidence regarding Foxp2 expression in the Tg DISC1 mouse model. In a postmortem human cohort study, increased expression of hippocampal FOXP2 was observed in schizophrenia subjects compared to controls, indicating that alteration in neural processes is influenced by FOXP2 protein^19^. Importantly, FOXP2 is associated with the ability to acquire spoken language in humans, while language deficit is considered one of the symptoms of schizophrenia. Consequently, the FOXP2 gene might be involved in the language disturbances found in patients with schizophrenia. Our results are consistent with the human research, suggesting that FOXP2 may have an important role in schizophrenia.

Our results further suggest that a combination of NAP with a standard of care RIS treatment can normalize FOXP2 altered expression, thereby protecting against brain dysfunction-related language disturbances which are associated with the negative symptoms of schizophrenia. Recent clinical studies identified a significant beneficial effect of NAP treatment on the UCSD Performance-based Skill Assessment– functional activity^33^ and enhancement of the neuro-pathological markers at the dorsolateral prefrontal cortex area in patients with schizophrenia^43^. Those studies further support our preclinical prediction and emphasize the need for additional investigations.

Future studies that focus on understanding the mechanism of action of NAP and RIS on the FOXP2 gene-altered expression are needed. Those studies may have significant clinical impact on the treatment of cognitive and negative alterations, characteristic for schizophrenia and other psychiatric disorders. Improvement of the results of long-term treatment and increase in the disease-related quality of life among patients with schizophrenia has an important economic and humane significance.

## Materials and Methods

### Drug selection

Bioinformatics were applied using a screening application on FDA approved drugs directories on Edesign web server^23^,^24^. A lead structure of NAP interacting motif SIP (SKIP)^8^, along with EB family members known as interacting agents with NAP was used for this purpose (PDB 3GJO)^44^, and 3TQ7^13^. Inclusion criteria of drugs was set according to the estimated characteristics of substances that are able to pass the blood-brain barrier (BBB): molecular weight under 400 g/mole, topological polar surface area (TPSA) under 60 and represent water/octanol partition coefficient and measure of hydrophobicity (xlogP) between 0.1 and 3.0^45^. Two docking applications were used for binding assessment (Swissdock^46^ and Patchdock^47^, comparing the energy binding level in different conformations.

### Sulfolink^®^ coupling gel-NAP affinity chromatography

Affinity chromatography was performed as before^8^, with SulfoLink® included Immobilization Kit for Peptides (Pierce-Biotechnology, USA) conjugated to NAP (CKKKGGNAPVSIPQ) loaded with recombinant EB3(3 mg) in the absence or presence of Risperidone-CRS(3 mg) (EP, Sigma-Oldaich). Sodium dodecyl sulfate – polyacrylamide gel electrophoresis (SDS–PAGE) was then performed followed by Coomassie Brilliant Blue (CBB) staining^25^,^48^.

### The Disc1 mouse model

The mutant hDISC1 mouse model was developed by Pletnikov and coworkers that made use of the Tet-off double transgenic system under the control of the CAMKII promoter. The two mutant lines are bred together in order to generate the double transgenic model mice. The responder line STOCK DISC1 Tg (Teto-DISC1*) 1001Plet/J; (The Jackson Laboratory, Bar Harbor, ME, USA) is a single transgenic line with inducible expression of mutant hDISC1 under the control of the tetracycline operator (tet-operator also called tetracycline-responsive element). The activator line CBA-Tg (Camk2a-tTA) 1Mmay/j mice express tTA driven by the calcium-calmodulin-dependent kinase II-α (CAMKII) promoter which produces a predominant expression of the transgene in forebrain neurons. Expression of mutant hDISC1 occurs when tetracycline-trans activator (tTA) binds to TRE and activates the promoter^7^. Expression of hDISC1 can be regulated by doxycycline (DOX) added to mouse food. DOX binds to tTA and prevents tTA from binding to TRE, leading to suppression of transcription of hDISC1^7^.

All animal procedures were conducted according to the guidelines of Tel Aviv University, Israel. The experiment was approved by the Animal Care and Use Committee of Tel Aviv University, under governmental permission.

### Dox treatment

A group of the inducible DISC1 (Tg) mice and a group of control littermates (WT) were subjected to doxycycline (DOX) treatment in order to turn off the expression of the inducible DISC1 mutant. 5–8-month-old mice received DOX in their drinking water (2 mg/ml). After four weeks of DOX exposure, NAP daily treatment was added to the drug administration regimen as described below.

### Drug tests

5–8-month-old mice (female and male) Tg and WT animals were subjected randomly to receive drug treatment by the following groups: vehicle (DD), NAP (davunetide), Risperidone (RIS), NAP and RIS combined solution (MIX). Vehicle (DD) treatment groups served as a control. Drug solutions were administered by the intranasal (I/N) route using pipette tips once a day (5 μl/each nostril) for a one-month period. Each substance administered was dissolved in DMSO 5 mg/ml and DDx5. DDx5 consisted of 7.5 mg/ml of NaCl, 1.7 mg/ml of citric acid monohydrate, 3 mg/ml of disodium phosphate dehydrate and 0.2 mg/ml of benzalkonium chloride solution (50%)^37^. NAP (Bachem – through Allon Therapeutics, USA and Canada) solution was given at a dose of 0.5 μg\5 μl\day\per mouse. RIS solution (Risperidone CRS, Sigma, France) was administered at a dose of 0.25µg\5µl \ per mouse. Control solution vehicle (DD) consisted on DDX1 (fivefold diluted DDx5) and DMSO 5 mg/ml and was applied by intranasal administration (I/N) at a dose of 5 μg\day. Following a month of daily treatments, the mice were subjected to behavioral tests (below) while continuing to receive daily treatments 2 h before the behavioral tests. All behavioral tests were performed between 9:00 am and 6:00 pm.

### Object recognition

The test is based on visual discrimination, the ability to identify and discriminate between the two different objects, which tests memory skills. The test was carried out as before^27^.The data was analyzed with the following formulas: D1 = b − a, when ‘a’ designated as the exploration time of the familiar object and ‘b’ designated as the exploration time of the novel object, and D2 = D1/(b + a). This formula evaluates the mouse capability to discriminate between the novel object and the familiar object^49^.

### Elevated plus-maze

The elevated plus maze trial is used for testing anxiety and is based on the assumption that animals suffer from fear of open spaces. The maze consisted of two open arms and two closed arms (50 cm × 10 cm × 40 cm each). The arms of each type are opposite to each other. The maze is elevated to 50 cm height from the floor. The experiment was conducted in a testing room with a dim light. Mice were placed onto the center of the maze, facing an open arm, and left free to explore it for 5 min. The time spent in the open and closed arms was registered and compared. The data was analyzed using the following formula: D2 = (b − a)/ (b + a), when ‘a’ designated the time spent in the open arms and ‘b’ designated the time spent in the closed arms^37^. The longer stay in the closed arms reflects increased anxiety-like behavior^50^,^51^.

### RNA and Protein extraction

RNA and proteins were extracted by the NucleoSpin® RNA/Protein (MACHEREY-NAGEL, Düren, Germany). The quantity and purity of RNA were determined by measuring the optical density at 260 nm with a spectrophotometer (NanoDrop Technologies, Wilmington, DE). Protein extraction Samples were maintained in PBS + 1% SDS solution at −20 °C.

### Reverse transcription and quantitative Real-Time PCR

Samples with the same amount of total RNA were used to synthesize single-strand cDNA using qScript™ cDNA Synthesis (Quanta BioSciences, Inc., Gaithersburg, MD) according to the manufacturer’s instructions. Primer pairs were designed using the primer 3 web interface (http://frodo.wi.mit.edu/primer3/) and synthesized by Sigma-Genosys (The Woodlands, TX).

Hprt: 5′GGATTTGAATCACGTTTGTGTC3′; 5′AACTTGCGCTCATCTTAGGC3′

Foxp2: 5′TGGATTGAATGTATGTGTGG3′; 5′CACGAAGACCTCAATGGTT3′

Real-Time PCR was performed using the PerfeCTa® SYBR® Green FastMix®, ROX™ (Quanta) and The StepOnePlus™ Real-Time PCR Sequence Detection System (Applied Biosystems) according to the default thermocycler program for all genes. Data was analyzed with Data Assist™ v2.1 Software (Applied Biosystems).

Real-time PCR reactions were carried using 300 nM of each forward and reverse primers, and 2 μl/5 cDNA samples, total of 10 μl reaction volume. At the RT-PCR assay, the comparative Ct method was used for quantification of transcripts. Threshold cycle (CT) is the fractional cycle number at which the fluorescence passes the threshold. The samples of interest are normalized to an appropriate endogenous house-keeping gene using ΔCT, the difference between CT values of the target gene to a house- keeping gene in a single sample. All transcripts were compared to the stable housekeeping transcript, hypoxanthine-guanine phosphoribosyl transferase (HPRT). Due to the exponential nature of PCR for linear values, Fold change for each target gene calculated using the formula: 2^–ΔCT^.

### Western blot analysis

Equal quantities of brain proteins (prepared as above) were loaded onto polyacrylamide gels (4–20%, Bio-Rad, Hempstead, UK) and subjected to Western blotting as before^52^, with rabbit DISC1 antibodies (Sigma) and mouse monoclonal actin antibodies (MP Biomedicals, Solon, OH) at 4 °C for 16 h. Secondary antibodies conjugated to horseradish peroxidase were obtained from Jackson ImmunoResearch (West Grove, PA). Signal density was detected with the DNR bioimaging systems MiniBIS pro (DNR Bio-Imaging Systems Ltd. Maale Hachamisha, Israel). Scanned results were quantified using the TINA-2.0 software (Raytest Straubenhardt, Germany).

### Statistical analysis

Data was analyzed with the SigmaPlot software (Chicago, IL, USA) for Windows using Two-way Analysis of Variance (ANOVA), followed by Pairwise Multiple Comparison Procedures [the Fisher’s least significant difference (LSD) method was used]. In two-way ANOVA, genotype and treatment were analyzed as factors. For all statistical analysis a p-value ≤ 0.05 was considered statistically significant.

## Additional Information

**How to cite this article**: Vaisburd, S. *et al.* Risperidone and NAP protect cognition and normalize gene expression in a schizophrenia mouse model. *Sci. Rep.* **5**, 16300; doi: 10.1038/srep16300 (2015).

## Supplementary Material

Supplementary Information
