# Supplementary material for: Risperidone and NAP protect cognition and normalize gene expression in a schizophrenia mouse model
Source: Sci Rep. 2015 Nov 10;5:16300. doi: 10.1038/srep16300 (PMC4639790; doi:10.1038/srep16300)

Risperidone and NAP protect cognition and normalize gene expression in a schizophrenia mouse model

Short title: Risperidone and NAP protect Foxp2 in DISC1 mice

Sinaya Vaisburd[1], Zeev Shemer[1], Adva Yeheskel[2], Eliezer Giladi[1] and Illana Gozes[1]**

[1]The Lily and Avraham Gildor Chair for the Investigation of Growth Factors; The Elton Laboratory for Neuroendocrinology; Department of Human Molecular Genetics and Biochemistry, Sackler Faculty of Medicine, Sagol School of Neuroscience and Adams Super Center for Brain Studies; [2]The Bioinformatics Unit, George S. Wise Faculty of Life Sciences, Tel Aviv University 69978, Israel

**Correspondence should be addressed to:

Illana Gozes, Ph.D.

Professor of Clinical Biochemistry; The Lily and Avraham Gildor Chair for

the Investigation of Growth Factors; Director, The Adams Super Center for Brain Studies and

The Edersheim Levie-Gitter fMRI Institute; Head, the Dr. Diana and Zelman Elton (Elbaum)

Laboratory for Molecular Neuroendocrinology

Sackler Faculty of Medicine

Tel Aviv University; Tel Aviv 69978, Israel

Phone: 972-3-640-7240; Fax: 972-3-640-8541

E-mail: igozes@post.tau.ac.il

Supplementary Information

Suppl. Figure 1. The DISC1-mutated mice do not show significant deficits in the Morris water maze.

The Morris water maze was carried out as before[1]. In short, mice received daily intranasal NAP for 2.5 months. Mice were subjected to two daily tests in a water maze, including a hidden platform. The size of the pool was 120X120 cm^2 and the size of the platform is 12X12 cm^2, every day for the first test, both the platform and the animal were situated in a new location with regards to the pool (with the pool being immobile). The experiment was performed as follows: the animal was positioned on the platform for 20 seconds and then placed in the water. The time

required to reach the platform (indicative of learning and intact reference memory) was measured (first test). After 20 second on the platform, the animal was placed back in the water (in the previous position) for a second test and search for the hidden platform (retained in the previous position). The time required to reach the platform in the second trial was recorded, indicative of short-term (working) memory. A maximal exploration time of 90 second per trial was allocated to each daily test. The trial was performed daily, on 5 consecutive days. A probe trial test that assesses spatial memory was used as follows. On the 5th day of testing, the platform was removed and the animals were subjected to swimming in the pool (for 90 seconds) without the platform; in these experiments, the time spent in the quadrant of the pool where the platform used to be was recorded, indicative of spatial memory. The last test included a visible platform trial that is performed 2 hours after the probe test. Measurements were performed with the HVS video tracking system (HVS Image Ltd., Hampton, UK).
Four experimental groups were compared, WT treated with vehicle (DD), WT-treated with NAP, Tg treated with DD and Tg treated with NAP (n=8/experimental group, except for the NAP-treated Tg group, which had 9 mice/group).
The results for the second daily trial (A) and the probe test (B) are shown.
(A) The second daily trial, showing latency to reach the hidden platform over 5 experimental days, showed no significant differences between the groups. The results indicate no differences in short-term spatial memory.
(B) The probe test, measuring spatial memory, showed no significant group differences.

Suppl. Figure 2. The DISC1-mutated mice do not show significant changes in activity in the open field.
Open field test was performed essentially as before. In short, after three weeks of daily NAP (or vehicle) treatment, the mice were individually placed in the center of an open field (50 cm ×50 cm square black arena) and left to explore freely for 120 min. The distance moved was recorded using the EthoVision pro video tracking system and software (Noldus Inc. Leesburg, VA). No significant differences were observed between the tested groups, including the same test groups outlined in Suppl. Fig. 1).

Suppl. Figure 3. The DISC1-mutated mice are socially impaired.
The main principle of the social recognition trial is based on the free choice of a subject mouse to spend time in one of three compartments during two experimental sessions, including an indirect contact with an unfamiliar mouse. In order to describe tested mouse social tendencies, the relative time period spent with containment cup hosting a novel mouse (preference for a novel mouse vs. empty cup) is measured. Typically, wild type animal will spend significantly more time in the compartment with novel mouse rather than the compartment with an empty cup, indicating a normal sociability, social motivation and affiliation. Data is expressed as a relative discrimination index D2= (time spent with novel mouse - time spent with an empty cup) /total duration at both chambers. Experiments were performed essentially as before [2]. Transgenic mice treated with DD showed a significant preference to the empty cup than the unfamiliar mouse, in contrast to control WT mice. There was a statistically significant difference in time spent with novel mouse/empty cup of WT DD treated mice and Tg DD -treated mice (discrimination index, 0.473 vs. 0.228; **P=0.005, for genotype effect). No significant differences were found in the discrimination index between all treatments within the transgenic mice; NAP treatment did not reach a significant result (p=0.168), however, unlike the Tg-DD group, the Tg-NAP group did

not differ from the control WT group, suggesting a potential effect of NAP, and the requirement for a larger study group for future evaluations.
Experiments were videotaped in a blinded fashion and the duration and number of direct (active) contacts between the subject mouse and the containment cup housing or not housing the unfamiliar mouse, for each chamber individually were counted for a 10 minute period. Data are expressed as mean (± SEM), of the factor D2= time spent with novel mouse-time spent with an empty cup/total duration at the right and left chambers. A significant difference was found between Tg under DD treatment and control WT mice under DD treatment (*p=0.005).

References

1. Merenlender-Wagner, A., Pikman, R., Giladi, E., Andrieux, A. & Gozes, I. NAP (davunetide) enhances cognitive behavior in the STOP heterozygous mouse--a microtubule-deficient model of schizophrenia. *Peptides* **31**, 1368-1373 (2010).
2. Malishkevich, A., *et al.* Activity-dependent neuroprotective protein (ADNP) exhibits striking sexual dichotomy impacting on autistic and Alzheimer's pathologies. *Transl Psychiatry* **5**, e501 (2015).

Suppl. Figure 1. The DISC1-mutated mice do not show significant deficits in the Morris water maze.

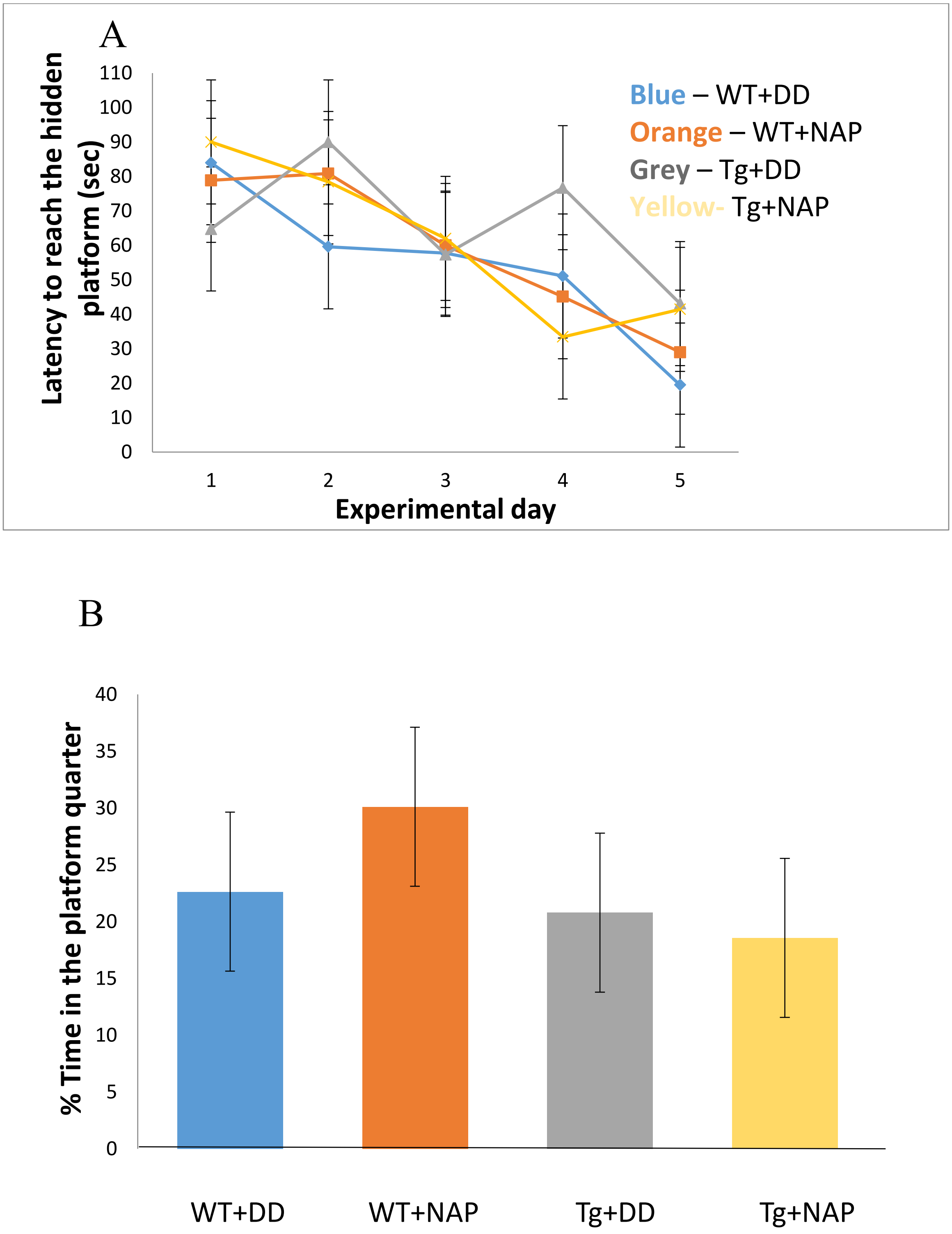

average	22.6	30.1	20.8	18.8
N	8	8	8	9

Suppl. Figure 2. The DISC1-mutated mice do not show significant changes in activity in the open field.

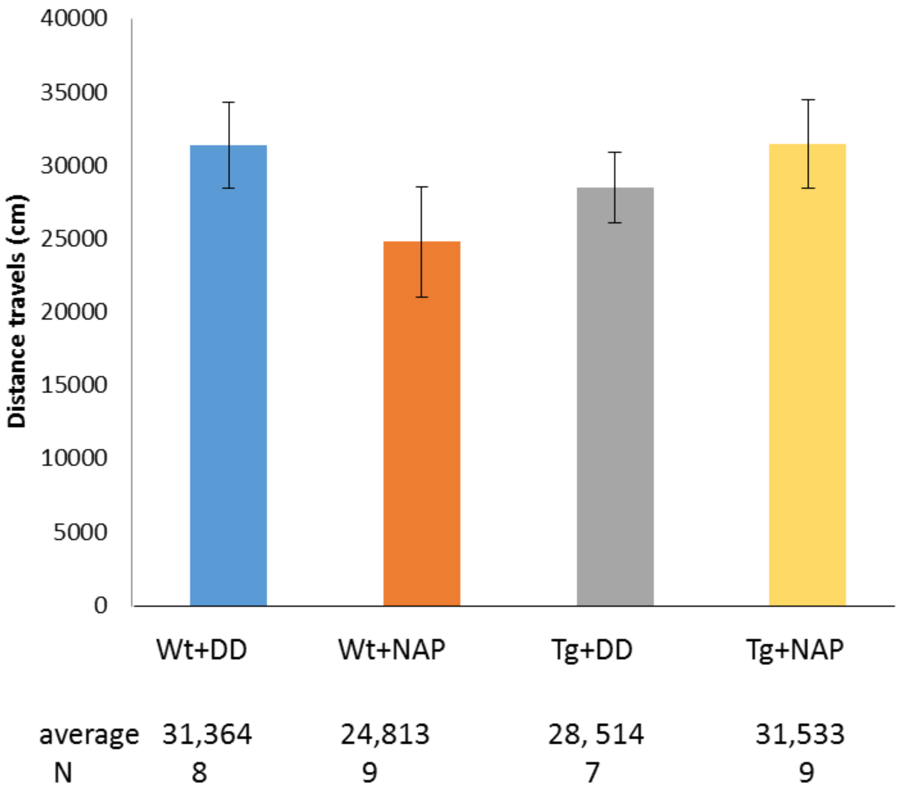

Suppl. Figure 3. The DISC1-mutated mice are socially impaired.

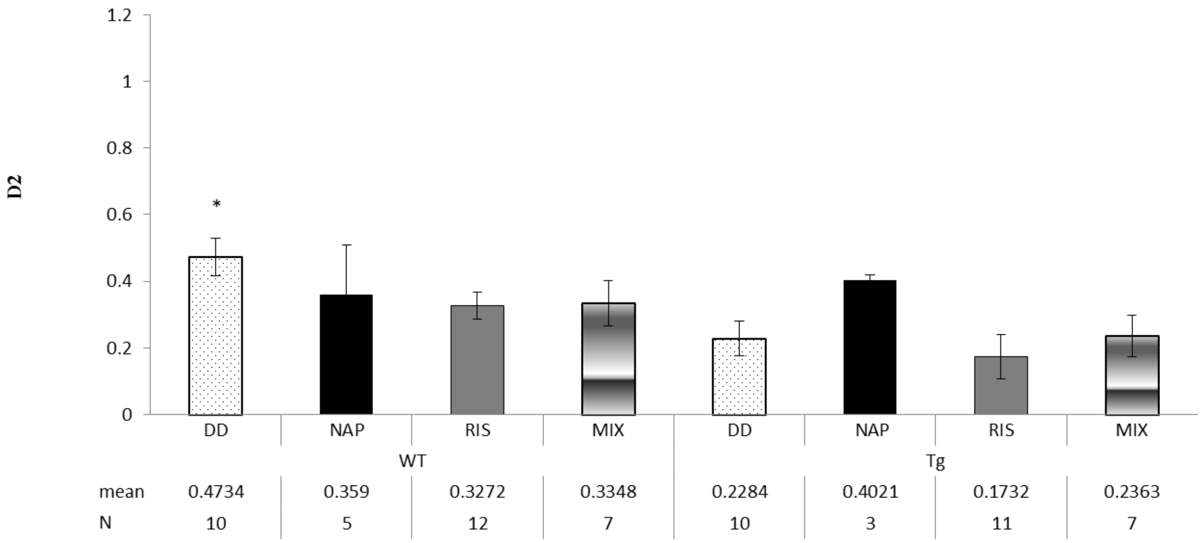

Supplement: Supplementary Information [file srep16300-s1.pdf]
